# Correlations between Fatty Acid Profile and Body Fat Distribution in Postmenopausal Women—A Cross Sectional Study

**DOI:** 10.3390/nu14183865

**Published:** 2022-09-18

**Authors:** Anna Maria Cybulska, Kamila Rachubińska, Karolina Skonieczna-Żydecka, Arleta Drozd, Jolanta Pawlik, Ewa Stachowska, Aneta Cymbaluk-Płoska, Elżbieta Grochans

**Affiliations:** 1 Department of Nursing, Pomeranian Medical University, Szczecin, Żołnierska 48, 71-210 Szczecin, Poland; 2 Department of Biochemical Science, Pomeranian Medical University, Szczecin, Broniewskiego 24, 71-460 Szczecin, Poland; 3 Department Human Nutrition and Metabolomics, Pomeranian Medical University, Szczecin, Broniewskiego 24, 71-460 Szczecin, Poland; 4 Institut für Nephrologie und Dialyse Salem-Spital, Schänzlistrasse 39, 3000 Bern, Switzerland; 5 Department of Gynecological Surgery and Gynecological Oncology of Adults and Adolescents, Pomeranian Medical University, Szczecin, 70-111 Szczecin, Poland

**Keywords:** menopause, fatty acid profile, body mass index, waist–hip ratio, body adipose index

## Abstract

The aim of the study was to assess the fatty acid profile of the whole blood of postmenopausal women, taking into account anthropometric parameters. The study involved 156 healthy women with an average age of 60 (SD = 6.3 years) years who were living in the West Pomerania Province (Poland). An original questionnaire was presented to all patients, conducting anthropometric measurements of them: weight, height, waist and hip circumference, body mass index (BMI), waist–hip ratio (WHR) and body adipose index (BAI), as well as an assessment of the fatty acid profile by employing gas chromatography. It has been observed that in menopausal women, the concentration of C16:1 increases with respect to their BMI (r = 0.205 *p* = 0.01). Similar correlations were noted with regard to body weight (C16:1 r = 0.177 *p* = 0.029). It was also shown that the concentration of C18trans11 (r = −0.166 *p* = 0.039), 18:2n6 (r = −0.165 *p* = 0.04) and n6/n9 (r = −0.194 *p* = 0.015) were negatively correlated with respect to their WHR, while the levels C16:1 (r = 0.22 *p* = 0.006), C18:1n9 (r = 0.22 *p* = 0.007), C24:1 (r = 0.251 *p* = 0.002), MUFA (r = 0.227 *p* = 0.046) and n9 (r = 0.224 *p* = 0.005) were correlated positively with respect to their BAI. The fatty acid profile of the whole blood of postmenopausal women is modulated to a poor extent by anthropometric variables. Therefore, more prospective research is warranted.

## 1. Introduction

For many years, scientists have been studying the effects of fatty acids (FA) on women’s health. There is evidence that in the body of a woman, there is a much higher concentration of docosahexaenoic acid (DHA) than there is in men, and this relationship is attributed to the effect of estrogens [1]. In addition, it has been observed that a skewed fatty acid profile influences the development of diseases such as obesity, overweight, metabolic syndrome and non-alcoholic fatty liver disease. Taking into account the risk of lipid metabolism disorders that are observed in postmenopausal women, which are mainly caused by a slowdown in energy metabolism, it seems important to assess the profile of their FA, which has been linked with the risk of noncommunicable diseases [2].

A review of the literature confirms the association between FA concentrations and a range of clinical variables in the menopausal period. Elevated levels of free fatty acids (FFA) in the plasma have been demonstrated to play a role in the pathogenesis of obesity, insulin resistance and cardiovascular diseases [3]. Jin et al. observed that *n*-3 PUFA levels in postmenopausal women are higher in those women that are using Menopausal Hormone Treatment (MHT). This relationship is probably due to the estrogen increase in the conversion of alpha-lipoic acid (ALA) to eicosapentaenoic acid (EPA) and docosahexaenoic acid (DHA) [4]. Similar results were obtained by Cybulska et al. [5] who showed that MHT is associated with a tendency (*p* = 0.053) to reduce the concentrations of oleic acid (C18:1*n*-9), arachidonic acid (C20:4) and all unsaturated fatty acids (*p* < 0.05). The longer that MHT was used, the higher the concentration of nervonic acid was (C24:1) (*p* = 0.04) and the lower the concentration of oleic acid (C18:2*n*-6) (*p* = 0.03) was. Research by Yammine et al. [6] demonstrated that women’s body mass indices (BMI) were positively correlated with women’s monounsaturated palmitoleic acid levels (r = 0.15, *p* = 0.01, q = 0.03). This study suggests that high blood levels of certain saturated fatty acids and monounsaturated palmitoleic acid in blood—possibly from both saturated FA consumption and endogenous lipogenesis—may have been associated with obesity in the Lebanese population. On the other hand, the studies of Micallef et al. [7] have showed that BMI, waist circumference and hip circumference were inversely correlated with *n*-3 Polyunsaturated Fatty Acids (PUFA), eicosapentaenoic acid (EPA) and docosahexaenoic acid (DHA) (*p* < 0.05 for all) levels in obese women. In Havel [8] and Lago et al.’s [9] studies, it was established that adipokines play an important role in lipid metabolism and might sustain inflammatory processes in the adipose tissue, and consequently, contribute to obesity and possibly to the metabolic syndrome. Moreover, it has been scientifically proven that the FA composition in serum lipid esters mirrored the dietary FA composition that has been ingested during last 6–8 weeks [10]. High levels of palmitic acid (16:0) and low levels of linoleic acid (18:2, *n*-6) in plasma lipid esters and a proportionally higher level of palmitoleic acid (16:1, *n*-7) are typical for people with insulin resistance and the metabolic syndrome [11].

The European Prospective Investigation into Cancer and Nutrition (EPIC) study showed that one of the causes of weight gain is having high levels of elaidic acid, the main industrial trans fatty acid [12]. With regard to omega-3 and omega-6 polyunsaturated fatty acids (*n*-3 and *n*-6 PUFA, respectively), scientific evidence has shown that the balance between these two groups may play an important role in the prevention and treatment of obesity. The excessive intake of omega-6 polyunsaturated fatty acids and a high omega-6/omega-3 ratio promotes the pathogenesis of many diseases, including cardiovascular disease, cancer, and inflammatory and autoimmune diseases, whereas, consuming increased levels of omega-3 PUFA (a lower omega-6/omega-3 ratio) exerts suppressive effects [13]. Excessive levels of circulating *n*-6 PUFA (high *n*-6/*n*-3 PUFA; ratios typical for Western diets) are associated with weight gain and obesity in both animal and human studies, while high levels of *n*-3 PUFA (lower *n*-6/*n*-3 PUFA) contribute favorably to an obesity phenotype [14]. Clinical and epidemiological studies indicate that there are many factors influencing body weight in postmenopausal women, and among them is FA. Therefore, we decided to evaluate the FA profile in a group postmenopausal women, regarding their anthropometric parameters.

We hypothesized that:

1. There are statistically significant correlations between the level of selected fatty acids and the anthropometric variables among perimenopausal women.
2. All obesity indices affect the levels of fatty acids in menopausal women.

We formulated the following research questions:

1. Are there statistically significant correlations between the level of fatty acids in the whole blood and the obesity index in perimenopausal women?
2. Is the fatty acid profile of postmenopausal women modulated by the obesity indices?

## 2. Materials and Methods

### 2.1. Participants

The study was approved by the Bioethical Commission of the Pomeranian Medical University in Szczecin (resolution number KB-0012/10/14). All subjects gave their written informed consent to participate in our study. Individuals were recruited by clinical referrals, newspaper advertisement, flyers and bulletin board notices in the medical centers that are located in the West Pomeranian Voivodeship, Poland.

We enrolled 156 Polish women of postmenopausal age, i.e., 60 years (SD = 6.3). Subjects were included if they:

- were at least one year from the last menstruation;
- had normal mammography results;
- had normal cervical smear results;
- had no clinically confirmed mental disease;
- were not receiving Menopausal Hormone Treatment;
- had normal blood pressure (less than 120 systolic and over 80 for diastolic);
- had a normal diet without supplementation of fatty acids (including omega-3), based on Polish cuisine;
- were Caucasian women residing in the West Pomeranian Voivodeship in Poland.

Subjects with neoplasms, diabetes (DM t 1, DM t 2 or women who had gestational diabetes mellitus), thyroid diseases, abnormal mammography results or a diagnosis of a mental health problem were excluded.

### 2.2. Measures

The data collection procedure involved a questionnaire to collect the basic information concerning the sociodemographic and health-related data. The participants provided information on: their education, place of residence, marital status, professional activity, menopausal status, as well as their use of stimulants and medications, especially those taken due to having hypertension, diabetes and coronary artery disease.

### 2.3. Procedures

Anthropometric data were collected (body weight, height and waist and hip circumference). For this purpose, a legalized medical scale was used with an integrated SECA 711 increase gauge, according to a standardized procedure with an accuracy of 0.1 kg and 0.1 cm, respectively.

On the basis of the obtained results, the body mass index was calculated from the formula, (BMI-body mass index): body mass [kg]/height [m])^2^. Based on the BMI value, the women were classified into an underweight group (BMI < 18.5), a normal weight group (18.5–24.9), an overweight group (25.0–29.9), and an I° obesity group (30.0–34.9), an obesity II° group (35.0–39.9) and an obesity III° group (BMI ≥ 40.0) [15].

Their waist and hip circumferences were measured to the nearest 0.01 m using a flexible steel metric tape (Seca). Waist circumference (WC) was defined as the horizontal distance around the abdomen at the umbilicus level. Hip circumference (HC) was measured as the distance passing horizontally through the two superior iliac bones. One of the indicators of abdominal obesity, the waist/hip ratio (WHR), was also determined. A value of more than 0.9 was considered abnormal, but we did not used this indicator for determining abdominal obesity [16].

Abdominal (central) obesity was defined as WC ≥ 80 cm (for European women) [17]. The BAI (body adipose index) was calculated from the formula, (BAI) = (hip circumference [cm]/height [m]^1,5^)-18.

Based on the BAI value, the women were classified as: underweight (<21%); healthy (21–33%), overweight (33–39%) or obese (>39%) [18].

### 2.4. Sampling and Fatty Acid Analysis

Blood was collected while the subjects had an empty stomach between 7:00 a.m. and 10:30 a.m. after a 10-min rest, in a sitting position, from the ulnar vein using a Vacutainer system by qualified nurses. Blood collection was performed in accordance with the applicable rules and procedures for the collection, storage, and transport of biological material. Blood was stored at −70 °C until the analysis began.

Fatty acids were isolated according to the Folch method [19]. Then, the methylation of the fatty acids was performed, and their content was analyzed based on a gas chromatography methodology using the Agilent Technologies 7890A GC System. The compounds were separated using a SUPELCOWAX™ 10 column Capillary GC Column (L × I.D. 15 m × 0.10 mm, df 0.10 μm) (Supelco, Cat. No: 24343).

The temperature was increased from 40 °C for 0.5 min, then increased at a rate of 25 °C/min up to 195 °C for 0 min, than 3 °C/min up to 205 °C for 0 min and 8 °C/min to 250 °C for 0.5 min; the total analysis time was 16.158 min, and the gas flow rate was 1 mL/min with nitrogen being used as the carrier gas. The qualitative and quantitative analysis were performed using ChemStation Software B.01.04 specialist software (Agilent Technologies, Cheadle, UK). The fatty acids were identified on the basis of the retention times that were predetermined for the respective standards (Sigma-Aldrich Canada, Oakville, ON, Canada and Neochema, Cayman Islands). The results were normalized using the internal standard C21:0 which was added to each test during isolation.

The blood was collected in accordance with the binding rules and procedures for the collection, storage and transport of biological material. The determination of the biochemical parameters was performed in a certified laboratory of the Pomeranian Medical University in Szczecin using standard commercial methods.

### 2.5. Statistical Analyses

The obtained results were used for a statistical analysis using the R program version 3.4.1 (R Foundation for Statistical Computing, Vienna, Austria, https://cran.r-project.org, accessed on 1 June 2022). The normality of the variable distribution was examined using the Shapiro-Wilk test. Consequently, continuous variables were expressed as medians and interquartile ranges. For the qualitative variables, a number was given and also expressed as a percentage. The differences in the sociodemographic and anthropometric variables were analyzed using the Mann-Whitney test. For the correlation analyses, Spearman’s/Pearson’s methods were used, as this was appropriate.

The significance level was assumed to be 0.05. To control for type I errors, the false discovery rate (FDR) approach was used. Post-hoc power analyses were conducted by means of G*Power software [20].

## 3. Results

### 3.1. Anthropometric Characteristics

For the purpose of our study, we included a group of 156 women with the mean age of 60 years (SD = 6.3). The mean time since they experienced menopause was 9 years (SD = 6.5). The subjects were divided into three groups, depending on BMI, WHR and BAI, according to WHO standards [15,16,17,18]. The first grouping was based on BMI (reference 25 kg/m^2^), abdominal obesity (reference WC = 80 cm) and BAI [healthy (BAI = 25–38%), overweight (BAI > 38–43%) and obese (BAI > 43%)], as shown in Table 1.

### 3.2. Analysis of the Fatty Acid Profile in Whole Blood

By analyzing the percentage of FA in the women that were enrolled, we found that the most prevalent FAs were: palmitic acid (C16:0, 24.61%; 448.10 µg/mL), linoleic acid (C18:2n6, 18.25%; 303.18 µg/mL), Omega 6 fatty acids (n6, 27.78%; 171.56 µg/mL) and saturated fatty acids (SFA, 46.49%; 860.56 µg/mL).

Among the isolated acids, a negligible amount of caprylic acid (C8: 0.0%), stearidonic acid (C18.4, 0%), erucic acid (C22.1cis13, 0%) and hexacosenoate acid (C26.0, 0%) were found. These fatty acids were not included in the further analysis. Raw data on the FA profile are presented in a Supplementary Table S1.

### 3.3. Analysis between BMI and FA Profile in Whole Blood

In our study the correlation between the BMI/body mass and the concentration of unsaturated fatty acids was assessed. We found that a poor positive correlation regarding palmitoleic acid (C16:1, *p* < 0.05) with BMI exists. There were no statistically significant correlations between BMI and other fatty acids. 

In the next stage of the analysis, the correlation between BMI and the concentration of: omega 3 fatty acids (*n*-3), omega 6 fatty acids (*n*-6), omega 9 fatty acids (*n*-9), saturated fatty acids (SFA), monounsaturated fatty acids (MUFA), unsaturated fatty acids (UFA), poly-unsaturated fatty (PUFA) and *n*-6: *n*-3, *n*-9: *n*-3, PUFA/SFA, MUFA/SFA and UFA/SFA ratios were estimated. These fatty acid contents were calculated as follows:

- The omega3 fatty acids (n3)—the sum of C18:3n3, C20:5, C22:5n3 and C22:6n3;
- The omega6 fatty acids (n6)—the sum of C18:2n6, C18:3n6, C20:4 and C22:4n6;
- The omega9 fatty acids (n9)—the sum of C18:1trans11, C22:1 cis13 and C24:1;
- The saturated fatty acid (SFA)—the sum of C10:0, C12:0, C14:0, C15:0, C16:0, C17:0, C18:0, C22:0 and C23:0;
- The monounsaturated fatty acid (MUFA)—the sum of C14:1, C16:1, C18:1n9, C18:1trans11 and C24:1;
- The polyunsaturated fatty acid (PUFA)—the sum of C18:2n6, C18:3n6, C18:3n3, C18:4, C20:4, C20:5, C22:4n6, C22:5n3 and C22:6n3;
- The unsaturated fatty acid is sum (UFA)—the sum of MUFA and PUFA.

A poor negative correlation regarding n6/n9 (*p* = 0.004) and BMI was demonstrated. However, when we look at the correlation of the median values of particular acid concentrations by the presence of obesity, we found no statistically sound associations. The statistics are presented in Table 2.

### 3.4. Analysis between FA in Whole Blood and WHR and WC

In the next stage of our analysis, we tried to look whether WHR correlates with the FAs. We found a poor negative correlation regarding trans-vaccenic acid (C18:1trans11) and linoleic acid (C18:2n6) (*p* < 0.05) and WHR. There was no statistically significant correlation between WHR and other fatty acids. We found a positive correlation between palmitoleic acid (C16:1) and WC (*p* < 0.05). There was no other statistically significant association between WC and other fatty acids, also there was none regarding a grouping by the WC parameter. The results are presented in Table 3.

We also analyzed the correlation between WHR and the concentration of omega 3 fatty acids (*n*-3), omega 6 fatty acids (*n*-6), omega 9 fatty acids (*n*-9), saturated fatty acids (SFA), monounsaturated fatty acids (MUFA), unsaturated fatty acids (UFA), poly-unsaturated fatty (PUFA) and *n*-6: *n*-3, *n*-9: *n*-3, PUFA/SFA, MUFA/SFA and UFA/SFA. We found a poor negative correlation regarding n6/n9 (*p* < 0.05). The results are presented in Table 3.

### 3.5. Analysis between BAI and FA Profile in Whole Blood

In the next stage of analyzes, a correlation between BAI and the FAs was evaluated. We also looked at the median values of the tested FAs in the groups according to the parameter of BAI (Healthy, Overweight and Obese). We found a positive correlation regarding palmitoleic acid (C16:1), oleic acid (C18:1n9), nervonic acid (C24:1), monounsaturated fatty acids (MUFA), omega 9 fatty acids (n9) and BAI (*p* < 0.05). In the obese women, the median concentration of palmitoleic acid (C16:1) was elevated when compared to that of the normal and overweight counterparts, whilst the content of oleic acid (C18:1n9) was significantly increased with the elevated status of an exceeding BAI. The results are presented in Table 4.

## 4. Discussion

One of the common problems for menopausal women is weight gain and the tendency to accumulate fat in the abdominal area. These changes often take place without clear changes in body weight, which makes it difficult to recognize the pathological changes in the accumulation of intra-abdominal fat [21,22]. The literature confirms the complexity and uniqueness of the menopausal period. Although menopause is common among women, each woman has a unique experience of menopause.

In our study, the correlation between the BMI/body mass and the concentration of unsaturated fatty acids was assessed. We found that a poor positive correlation regarding palmitoleic acid (C16:1, *p* < 0.05) with BMI exists. There were no statistically significant correlations between BMI and other fatty acids. Moreover, a poor negative correlation regarding omega 6 fatty acids/omega 9 fatty acids (n6/n9, *p* = 0.004) and BMI was demonstrated. However, when we look at the median values of particular acid concentrations by the presence of obesity, we found no statistically sound associations. Moreover, we found a poor negative correlation regarding trans-vaccenic acid (C18:1trans11) and linoleic acid (C18:2n6) (*p* < 0.05) and WHR. We observed a positive correlation between palmitoleic acid (C16:1) and WC (*p* < 0.05). There was no other statistically significant association between WC and other fatty acids, also there was none regarding the grouping by the WC parameter. We found a positive correlation regarding palmitoleic acid (C16:1), oleic acid (C18:1n9), nervonic acid (C24:1), monounsaturated fatty acids (MUFA), omega 9 fatty acids (n9) and BAI (*p* < 0.05). In the obese women, the median concentration of palmitoleic acid (C16:1) was elevated when compared to that of the normal and overweight counterparts, while the content of oleic acid (C18:1n9) was significantly increased with the elevated status of an exceeding BAI.

There are few studies assessing the FA profile of postmenopausal women using anthropometric parameters. Most often, scientists evaluate the effect of MHT use on fatty acid levels. In our previous publication [5], we showed that women receiving sex hormones had elevated tetradecanoic acid (C14:0) and palmitic acid (C16:0) levels, while their levels of oleic acid (C18:1*n*-9) and arachidonic acid (C20:4) were decreased, compared to those women who did not receive MHT. Hormone therapy significantly lowered the content of all saturated fatty acids [5]. SFA levels were significantly increased in women receiving MHT, and there were no differences in the omega 9 fatty acids (*n*-3) levels between the subgroups. Similar results were presented by Stark et al. [23] and Ottosson et al. [24].

A review of the literature shows that many scientists analyze the differences in fatty acid levels with regard to anthropometric parameters between men and women.

Women have more body fat than men, and FFA kinetics is sex-differentiated [25]. Research by Arner et al. [26] showed that the levels of FFA in women were slightly higher than they were in men, but the effect of obesity was similar for both sexes. The researchers did not record information on the status of menopause, but instead they collected data to suggest that menopause did not have a major impact on the observed differences in FFA levels. It was noted that FFA levels were significantly higher in obese women when they were compared to non-obese women in the age groups <45 or >55 years (*p* < 0.0001). In addition, it was observed that the concentrations of free fatty acids (FFA) were almost 30% higher in obese patients, compared to those of non-obese patients.

In turn, studies by Warensjo et al. [27] showed positive correlations between obesity markers (BMI, HOMA-IR) and the level of certain acids: palmitic acid (C16:0), palmitoleic acid (C16:1), stearic acid (C18:0), α-Linolenic acid (C18:3) and mead acid (C20:3).

These relationships were independent of age and physical activity. It was found that women have significantly higher delta9 levels and lower delta-6 desaturase enzyme activity levels than men do.

Research by Yammine et al. [6] showed that BMI was significantly positively correlated with the total amount of saturated fatty acids (SFA) in both men (r = 0.40, *p* < 0.0001, q < 0.0001) and women (r = 0.33, *p* < 0.0001, q < 0.0001). For individual saturated fatty acids (SFAs), there was no significant correlation between BMI or waistline and odd fatty acids, pentadecanoic acid (C15:0) and heptadecanoic acid (C17:0). It is worth noting that in these studies, especially among women, BMI was significantly positively correlated with monounsaturated fatty acids (MUFA, r = 0.15, *p* = 0.01, q = 0.03). Moreover, among women, a negative correlation was found between BMI and the sum of the trans fatty acids that were recorded (TFA, r = −0.17, *p* = 0.007, q = 0.03). When distinguishing individual trans fatty acids (TFA) isomers, different correlations were found with BMI, depending on sex, with elaidic acid (r = −0.14, *p* = 0.02, q = 0.05) and linoleic acid (r = −0.15, *p* = 0.01, q = 0.03) showing significant inverse correlations in women, while trans-α-linolenic acid showed a positive trend in men (r = 0.22, *p* = 0.01, q = 0.10). According to Paniagua et al. [28] MUFA-rich diets prevent a postprandial decline in the peripheral adiponectin gene expression. Adiponectin is one of the cytokines that is secreted by adipose tissue, regulating energy metabolism through interactions with glucose and insulin, stimulating fatty acid oxidation, lowering plasma triglycerides and improving glucose metabolism by increasing insulin sensitivity [29].

In the research of Yammine et al., no significant correlation was found between *n*-6 PUFA, *n*-3 PUFA or the ratio of *n*-6/*n*-3 PUFA to BMI, waist circumference, and percentage of body fat. On the other hand, divergent correlations with regard to sex were found between individuals’ *n*-6 and *n*-3 PUFAs and BMI values. In men, *n*-3 α-linolenic acid, an essential fatty acid from the *n*-3 family, tended to be positively correlated with obesity (r = 0.18, *p* = 0.04, q = 0.24), while *n*-3 docosahexaene (DHA) was negatively correlated with it (r = −0.24, *p* = 0.009, q = 0.10). In women, *n*-6 γ-linoleic acid (r = 0.17, *p* = 0.006, q = 0.03) and *n*-3 eicosapentaenoic acid (EPA, r = 0.14, *p* = 0.02, q = 0.05) were positively correlated with BMI. All individual fatty acids that were significantly correlated with obesity indices showed a significant linear relationship with BMI and waistline. There was no statistically significant linear relationship between BMI or waistline and all other individual fatty acids such as pentadecanoic acid (C15:0), heptadecanoic acid (C17:0) and most of omega 6 fatty acids (*n*-6) and *n*-3 PUFAs.

Similar results for total saturated fatty acids (SFA) and BMI were presented in the studies by Aglago E.K et al. [30]. Moreover, in these studies, high relative serum levels of dihomo-γ-linolenic acid (DGLA) were associated with obesity, adipose tissue accumulation and waist circumference. In addition, the publication by Hammand et al. [31] indicates that saturated fatty acid (SFA) consumption leads to increased body obesity. Similar results were obtained among the women that were included in the Nurses’ Health study, which confirmed a significant correlation between SFA consumption and the accumulation of abdominal fat in women [32]. In addition, it has been shown that the positive correlations between total saturated fatty acids (SFA) and BMI or waist circumference are likely to be due to the presence of stearic acid in women. In contrast, among the SFAs with odd chains that are derived from dairy products, heptadecanoic acid showed an insignificant inverse relationship with BMI and waist circumference in women. These data suggest that individual SFAs may have varying effects on obesity, depending on their food sources and endogenous synthesis.

Studies by Del Pozo et al. [33] showed that BMI was positively correlated with the relative concentration of saturated fatty acids (SFA) (β = 0.94, Q-Val = 0.001) and palmitoleic, dihomo-γ-linolenic (DGLA), arachidonic (AA) and α-linolenic acids. In contrast, they are negatively correlated with oleic, gondoic, trans-vaccenic, linoleic and γ-linolenic acids. Total fat percentage was positively related to DGLA and AA, and it was negatively related to linoleic and γ-linolenic acids. Low relative concentrations of some SFAs and high levels of *n*-6 PUFAs have been associated with a greater waist circumference.

Many studies confirm a positive relationship between palmitoleic acid or adipose tissue and obesity [34,35,36,37,38]. The research by Kurotani et al. [39], which was conducted among Japanese workers, showed that high serum palmitic acid levels led to elevated C-peptide levels, insulin resistance and inflammation that are factors associated with obesity.

Studies by Smit et al. [40] showed a significant discrepancy between the relationship of saturated fatty acids trans fatty acids (TFA) isomers and obesity. The studies have shown a relationship between C18:2 fatty acids and BMI, waist circumference and skinfold thickness (*p* < 0.01 for each relationship). Inverse relationships were found for elaidic acid (C18:1), and BMI and waist circumference (*p* < 0.0001). This study suggests that different trans fatty acid isomers may have different effects on obesity. Linolelaidic acid (C18:2) fatty acids show consistently positive associations with obesity measures.

In the studies of Hastert et al. [41] which were conducted in the American Multi-Ethnic Study of Atherosclerosis (MESA) cohort, no clear relationship was observed between the plasma phospholipid levels of all of the trans fatty acids and the baseline changes in BMI or BMI (during 10 years of observation). Different results were obtained during a 5-year follow-up in the European EPIC cohort, where the high baseline levels of elaidic acid and trans fatty acids (TFA) in the blood were found to be associated with an increased risk of weight gain [42]. Research by James et al. [43] showed that *n*-6 and *n*-3 PUFAs may have different effects on the development of obesity through their different influence on inflammation.

The significant discrepancies between the presented studies may be a consequence of differences in the methodology that was used of collecting the anthropometric data and the FA evaluations and the population characteristics. Therefore, further studies on the association between lipids and obesity indices in women at various stages of life, especially those in the perimenopausal period, seem to be important.

## 5. Limitations

We are aware of limitations in our study. These are as follows:

- Firstly, it is a cross-sectional study, therefore, we cannot fully assess the causal relationship between the fatty acids and the selected anthropometric parameters which was indeed noted by the reviewer;
- The FA levels were measured in the full blood, not in the RBC—the latter one reflects better the ingestion of fats with food;
- There is a limited power for this study, and larger sample size is warranted; we, however, checked the post-hoc power for significant correlations, and the power varied between 0.54–0.89;
- Menopause was determined by history, not estrogen levels, which is consistent with the WHO guidelines. The mean time from the last menstruation among the surveyed women was 9 years (SD = 6.5). Many studies examining various variables in postmenopausal women do not (if the goal does not require it) assess the level of hormones. Our study was also not intended to assess the effect of sex hormone levels on FAs, so it was not necessary to test for sex hormone levels;
- We have not analyzed the exact data on dietary habits using a standardized questionnaire, however, vegetarians and vegans were excluded from the study.

## 6. Conclusions

The fatty acid profile in postmenopausal women is modulated to a poor extent by anthropometric variables.

## Figures and Tables

**Table 1:** Anthropometric characteristics with a WC, BMI and BAI division.

Variables	BMI					WC					BAI							All Women	
	Normal Weight (*n* = 62)		Obese/Overweight (*n* = 94)		*p*	WC < 80 cm (*n* = 38)		WC ≥ 80 cm (*n* = 118)		*p*	Healthy (*n* = 87)		Overweight (*n* = 50)		Obese (*n* = 19)		*p*		
	Me	IQR	Me	IQR		Me	IQR	Me	IQR		Me	IQR	Me	IQR	Me	IQR		Me	IQR
Age [years]	58	7	59	7.25	0.104	58.5	5.25	59	9	0.338	58	7	59	6.25	62	8	0.0147	59	7.8
Height [cm]	161.5	9.75	162.5	8	0.813	160	11.25	162	9	0.339	164	8	158	7.5	160	6	<0.0001	162	9
Weight [kg]	61	7.5	75.5	13.5	<0.0001	61	8.25	73	16	<0.0001	65	12	73	17.75	89	12	<0.0001	70	16.5
BMI [kg/m^2^]	23.23	1.65	28.8	5.1	<0.0001	23.23	2.46	27.7	5.7	<0.0001	24.38	3.19	28.67	3.96	33.2	2.98	<0.0001	26.1	6.0
WC [cm]	80	8	91.5	15.25	<0.0001	76	5	90	13.25	<0.0001	82	10	90	17	102	11	<0.0001	85	15
HC [cm]	98	7.25	108.5	11	<0.0001	96	6.75	105.5	10.25	<0.0001	101	8	107.5	8.5	120	13	<0.0001	104	12.5
WHR [cm]	0.81	0.07	0.83	0.08	<0.0001	0.78	0.069	0.84	0.07	<0.0001	0.82	0.077	0.83	0.07	0.84	0.107	<0.0001	0.8	0.07
WHtR [cm]	0.49	0.05	0.57	0.09	0.407	0.47	0.04	0.55	0.09	0.403	0.5	0.063	0.57	0.85	0.62	0.073	0.0638	0.53	0.1
BAI [%]	29.65	4.0	35.05	6.32	<0.0001	30.05	4.64	33.61	7.09	<0.0001	30.03	3.18	35.62	2.85	42.42	2.94	<0.0001	32.5	6.4

BMI—body mass index, WC—waist circumference, BAI—body adipose index, WHtR—waist–height ratio, WHR—waist–hip ratio, HC—hip circumference, Me—median, IQR—interquartile range, *p*—significance level.

**Table 2:** Concentrations of fatty acids in postmenopausal women in terms of BMI by means of a Spearman’s correlation coefficient.

Fatty Acid (%)	BMI [kg/m^2^]			Body Mass ^#^ [kg]			BMI ^^^					
							Normal Weight		Obese/Overweight		*p*	FDR
	r	*p*	FDR	r	*p*	FDR	Me	IQR	Me	IQR		
C10:0	0.096	0.231	0.71	0.080	0.322	0.58	1.52	1.14	1.71	1.14	0.356	0.98
C12:0	0.045	0.577	0.73	0.099	0.222	0.58	0.24	0.085	0.25	1.112	0.669	0.98
C14:0	0.08	0.32	0.72	0.143	0.075	0.52	1.18	0.653	1.34	0.767	0.633	0.98
C14:1	0.024	0.769	0.82	0.025	0.825	0.90	0.11	0.140	0.11	0.11	0.951	0.98
C15:0	−0.077	0.34	0.72	−0.058	0.461	0.72	0.33	0.188	0.34	0.184	0.892	0.98
C16:0	0.041	0.61	0.73	0.103	0.202	0.58	24.16	4.63	24.52	5.510	0.938	0.98
C16:1	0.205	0.01 ^1^	0.19	0.177	0.029 ^2^	0.52	1.19	0.537	1.27	0.827	0.125	0.98
C17:0	−0.058	0.473	0.73	−0.002	0.964	0.98	0.4	0.145	0.42	0101	0.944	0.98
C18:0	0.009	0.908	0.91	0.057	0.487	0.72	11.84	13.250	12.64	13.00	0.999	0.999
C18:1n9	0.111	0.168	0.69	−0.011	0.888	0.94	16.35	4.777	17.03	5.306	0.347	0.98
C18-1trans11	−0.035	0.666	0.73	−0.078	0.324	0.58	1.55	0.578	1.51	0.618	0.852	0.98
C18:2n6	−0.121	0.133	0.69	−0.127	0.110	0.52	17.87	6.385	18.08	6.963	0.338	0.98
C18:3n6	0.104	0.196	0.69	0.076	0.358	0.61	0.26	0.192	0.29	0.169	0.397	0.98
C18:3n3	−0.069	0.393	0.73	−0.090	0.257	0.58	0.57	0.228	0.55	0.271	0.776	0.98
C20:4	−0.073	0.365	0.73	−0.123	0.123	0.52	8.32	3.293	8.10	3.486	0.442	0.98
C20:5	0.016	0.839	0.86	0.026	0.757	0.86	1.2	0.853	1.15	0.841	0.415	0.98
C22:0	−0.092	0.251	0.71	−0.087	0.093	0.52	0.31	0.465	0.26	0.422	0.392	0.98
C22:4n6	−0.039	0.631	0.73	−0.057	0.473	0.72	0.79	0.44	0.78	0.414	0.529	0.98
C22:5n3	−0.035	0.665	0.73	−0.044	0.581	0.76	1.23	0.502	1.23	0.590	0.787	0.98
C22:6n3	−0.103	0.202	0.69	−0.083	0.298	0.58	2.66	1.23	2.62	1.476	0.931	0.98
C23:0	0.036	0.659	0.73	0.064	0.687	0.85	0.15	0.26	0.16	0.294	0.918	0.98
C24:1	0.084	0.298	0.72	0.306	0.222	0.58	0.00	0.00	0.00	0.00	0.678	0.98
SFA	0.039	0.633	0.73	0.091	0.256	0.58	41.44	18	41.5	16.8	0.906	0.98
MUFA	0.144	0.073	0.69	0.029	0.721	0.85	19.43	5.53	20.2	6.2	0.267	0.98
PUFA	−0.112	0.163	0.69	−0.124	0.120	0.52	35.6	11.8	32	12.8	0.541	0.98
UFA	−0.039	0.633	0.73	−0.087	0.272	0.58	58.3	17.7	57.8	16.7	0.941	0.98
n3	−0.051	0.53	0.73	−0.052	0.513	0.73	5.7	2.4	5.8	2.8	0.558	0.98
n6	−0.117	0.144	0.69	−0.136	0.090	0.53	28.6	10.2	27	11	0.334	0.98
n9	0.122	0.13	0.69	0.03	0.976	0.98	16.3	4.6	17.4	5.4	0.298	0.98
n6/n9	−0.229	0.004 ^3^	0.15	−0.141	0.077	0.52	1.7	0.42	1.7	0.4	0.303	0.98
n6/n3	−0.039	0.627	0.73	−0.048	0.546	0.74	4	1.65	4.5	1.4	0.902	0.98
PUFA/SFA	−0.081	0.316	0.72	−0.112	0.161	0.58	0.89	0.54	0.79	0.54	0.696	0.98
MUFA/SFA	0.065	0.419	0.73	−0.03	0.705	0.85	0.48	0.26	0.49	0.3	0.550	0.98
UFA/SFA	−0.039	0.633	0.73	−0.089	0.269	0.58	1.41	0.83	1.39	0.8	0.920	0.98

r—correlation coefficient, *p*—significance level, Me—median, IQR—interquartile range; # Spearman’s rank ^. Mann–Whitney U; BMI—body mass index, FDR—false discovery rate. ^1,2^ Power 0.73. ^3^ Power 0.82.

**Table 3:** Concentrations of fatty acids in postmenopausal women in terms of WHR by means of a Spearman’s correlation coefficient.

Fatty Acid (%)	WHR			WC [cm] ^#^			WC ^^^					
							WC < 80		WC > 80		*p*	FDR
	r	*p*	FDR	r	*p*	FDR	Me	IQR	Me	IQR		
C10:0	0.048	0.551	0.78	0.109	0.179	0.58	1.52	1.39	1.78	1.32	0.567	0.74
C12:0	0.077	0.338	0.64	0.100	0.221	0.58	0.24	0.113	0.25	0.111	0.244	0.66
C14:0	0.133	0.098	0.28	0.121	0.134	0.58	1.13	0.598	1.30	0.757	0.164	0.66
C14:1	−0.066	0.411	0.73	−0.06	0.515	0.70	0.10	0.086	0.11	0.137	0.551	0.74
C15:0	−0.016	0.844	0.96	−0.02	0.793	0.79	0.32	0.184	0.34	0.187	0.460	0.71
C16:0	0.141	0.079	0.28	0.09	0.268	0.58	23.27	5.504	26.64	5.21	0.340	0.70
C16:1	0.063	0.432	0.73	0.205	0.011 ^1^	0.38	1.17	0.476	1.29	0.781	0.185	0.65
C17:0	0.021	0.792	0.94	0.033	0.697	0.75	0.40	0.096	0.42	0.118	0.633	0.80
C18:0	0.118	0.143	0.34	0.039	0.697	0.75	11.76	13.386	12.37	13.12	0.782	0.83
C18:1n9	−0.003	0.968	0.999	0.091	0.263	0.58	15.95	4.475	17.15	5.242	0.252	0.66
C18-1trans11	−0.166	0.039 ^2^	0.28	−0.052	0.509	0.70	1.57	0.525	1.51	0.652	0.758	0.83
C18:2n6	−0.165	0.04 ^3^	0.28	−0.151	0.057	0.58	20.57	20.57	17.87	6.507	0.060	0.66
C18:3n6	0.089	0.271	0.54	0.147	0.072	0.58	0.22	0.221	0.28	0.170	0.216	0.66
C18:3n3	0.02	0.801	0.94	−0.048	0.536	0.70	0.57	0.191	0.57	0.268	0.510	0.72
C20:4	−0.122	0.129	0.34	−0.075	0.343	0.58	8.49	3.608	8.04	3.197	0.401	0.70
C20:5	−0.052	0.52	0.77	−0.012	0.677	0.75	1.18	0.986	1.17	0.797	0.703	0.83
C22:0	−0.052	0.522	0.77	−0.035	0.311	0.58	0.32	0.414	0.28	0.473	0.986	0.99
C22:4n6	−0.002	0.977	0.999	0.035	0.677	0.75	0.77	0.436	0.79	0.412	0.843	0.87
C22:5n3	−0.146	0.069	0.28	−0.074	0.344	0.58	1.37	0.633	1.21	0.497	0.134	0.66
C22:6n3	−0.092	0.253	0.54	−0.120	0.130	0.58	2.77	1.477	2.59	1.293	0.380	0.70
C23:0	−0.025	0.761	0.94	0.09	0.46	0.68	0.13	0.242	0.17	0.299	0.284	0.69
C24:1	0.035	0.661	0.90	0.28	0.44	0.68	0.00	0	0.00	0	0.483	0.71
SFA	0.134	0.094	0.28	0.077	0.33	0.58	39.3	17.8	42	17.2	0.430	0.70
MUFA	0	0.999	0.999	0.113	0.160	0.58	19	5.4	20.2	6.2	0.193	0.66
PUFA	−0.149	0.063	0.28	−0.140	0.081	0.58	37	12.7	32	12.1	0.117	0.66
UFA	−0.134	0.094	0.28	−0.076	0.343	0.58	59.9	17.3	57.5	17.1	0.377	0.70
n3	−0.115	0.152	0.34	−0.105	0.190	0.58	5.9	3.1	5.7	2.4	0.729	0.83
n6	−0.156	0.052	0.28	−0.133	0.098	0.58	31.2	10.7	26.5	10.3	0.109	0.66
n9	−0.007	0.929	0.999	0.096	0.234	0.58	16	4.6	17.2	4.8	0.193	0.66
n6 /n9	−0.194	0.015 ^4^	0.28	−0.035	0.665	0.75	1.9	0.56	1.72	0.4	0.002	0.07
n6 /n3	−0.031	0.705	0.92	0.024	0.762	0.79	4.6	2.4	4.6	1.5	0.336	0.70
PUFA/SFA	−0.145	0.071	0.28	-.0117	0.144	0.58	0.96	0.56	0.78	0.53	0.171	0.66
MUFA/SFA	−0.06	0.457	0.74	0.031	0.703	0.75	0.49	0.27	0.48	0.29	0.704	0.83
UFA/SFA	−0.134	0.094	0.28	−0.074	0.357	0.58	1.53	0.82	1.37	0.79	0.418	0.70

r—correlation coefficient, *p*—significance level, Me—median, IQR—interquartile range; # Spearman’s rank ^ Mann–Whitney U; WC—waist circumference, WHR—waist–hip ratio, FDR—false discovery rate. ^1^ Power 0.73. ^2^ Power 0.55. ^3^ Power 0.54. ^4^ Power 0.68.

**Table 4:** Concentrations of fatty acids in postmenopausal women in terms of BAI by means of a Spearman’s correlation coefficient.

Fatty Acid (%)	BAI			BAI							
				Healthy (*n* = 87)		Overweight (*n* = 50)		Obese (*n* = 19)		*p*	FDR
	r	*p*	FDR	Me	IQR	Me	IQR	Me	IQR		
C10:0	0.132	0.1	0.46	1.7	1.3	1.5	1.3	2.2	1.7	0.15	0.44
C12:0	−0.006	0.9	0.92	0.25	0.1	0.2	0.1	0.3	0.13	0.6	0.76
C14:0	−0.04	0.6	0.78	1.3	0.7	1.2	0.8	1.3	0.8	0.2	0.44
C14:1	0.054	0.6	0.78	0.1	0.1	0.1	0.2	0.14	0.11	0.1	0.38
C15:0	−0.101	0.2	0.62	0.3	0.2	0.3	0.2	0.34	0.23	0.4	0.62
C16:0	−0.05	0.5	0.78	25	5	23.9	5.1	23.2	6.3	0.2	0.44
C16:1	0.22	0.006 ^1^	0.06	1.2	0.6	1.2	0.6	1.7	1	0.006	0.05
C17:0	−0.07	0.4	0.78	0.4	0.1	0.4	0.1	0.4	0.06	0.8	0.85
C18:0	−0.06	0.47	0.78	12.1	13.8	12.6	11.5	11.2	10.1	0.3	0.51
C18:1n9	0.22	0.007 ^2^	0.06	15.8	4.7	17.1	5.3	18.4	1.6	0.003	0.03
C18-1trans11	0.095	0.24	0.63	1.5	0.6	1.5	0.5	1.7	0.7	0.2	0.44
C18:2n6	−0.078	0.3	0.73	17.9	6.9	18.6	5.4	15.9	9.2	0.5	0.65
C18:3n6	0.103	0.2	0.62	0.26	0.17	0.28	0.17	0.33	0.12	0.04	0.23
C18:3n3	−0.1	0.2	0.62	0.57	0.21	0.58	0.29	0.5	0.3	0.3	0.51
C20:4	0.03	0.7	0.85	8.3	3.4	8.3	3.7	9	2.6	0.5	0.65
C20:5	0.011	0.9	0.92	1.2	0.9	1.2	0.9	1.1	0.6	0.8	0.85
C22:0	0.025	0.8	0.83	0.28	0.42	0.3	0.5	0.3	0.4	0.8	0.85
C22:4n6	0.073	0.4	0.78	0.7	0.4	0.8	0.5	0.9	0.2	0.1	0.38
C22:5n3	0.05	0.6	0.79	1.21	0.5	1.2	0.6	1.4	0.5	0.2	0.44
C22:6n3	−0.09	0.24	0.63	2.7	1.2	2.6	1.5	2.5	1.4	0.5	0.65
C23:0	0.072	0.4	0.78	0	0	0.14	0.25	0.3	0.4	0.3	0.51
C24:1	0.251	0.002 ^3^	0.06	0.74	0.4	0	0	0	0.2	0.9	0.93
SFA	−0.40	0.619	0.78	42.1	18.3	41.1	15.7	40.9	17.55	0.251	0.50
MUFA	0.227	0.046 ^4^	0.31	18.9	5.8	20.2	5	22.48	2.73	0.002	0.03
PUFA	−0.041	0.607	0.78	32.1	13.4	33.8	12.1	33.5	12.45	0.491	0.65
UFA	0.043	0.595	0.78	57	18.1	58.2	15.2	58.32	17.28	0.205	0.44
n3	−0.027	0.734	0.86	5.9	2.7	5.9	2.7	5.8	2.6	0.623	0.76
n6	−0.051	0.53	0.78	27.1	11	28.95	9.9	28.5	11.2	0.700	0.82
n9	0.224	0.005 ^5^	0.06	15.8	4.4	17.2	5.3	18.7	1.98	0.002	0.03
n6/n9	−0.149	0.0628	0.31	1.8	0.47	1.64	0.41	1.57	0.32	0.09	0.38
n6/n3	0.019	0.814	0.89	4.4	1.9	4.5	1.3	4.49	1.31	0.995	0.995
PUFA/SFA	−0.008	0.916	0.92	0.8	0.6	0.81	0.55	0.868	0.536	0.366	0.59
MUFA/SFA	0.153	0.0575	0.31	0.5	0.3	0.501	0.265	0.556	0.255	0.02	0.14
UFA/SFA	0.043	0.595	0.78	1.4	0.8	1.41	0.77	1.425	0.802	0.196	0.44

Me—median, IQR—interquartile range, BAI—body adipose index, FDR—false discovery rate. ^1,2^ Power 0.79. ^3^ Power 0.89. ^4^ Power 0.82. ^5^ Power 0.81.

## Data Availability

Not applicable.

## Supplementary Materials

The following supporting information can be downloaded at: https://www.mdpi.com/article/10.3390/nu14183865/s1, Table S1. The fatty acid profile in whole blood study group.
