# Supplementary material for: Correlations between Fatty Acid Profile and Body Fat Distribution in Postmenopausal Women—A Cross Sectional Study
Source: Nutrients. 2022 Sep 18;14(18):3865. doi: 10.3390/nu14183865 (PMC9502591; doi:10.3390/nu14183865)
Supplement: Supplementary file 1 [file nutrients-14-03865-s001.zip › nutrients-1890949-supplementary.pdf]

Table S1. The fatty acid profile of postmenopausal women

Fatty Acid		Fatty Acid (%)			Fatty Acid (ug/mL)		
		Mean	Min	Max	Mean	Min	Max
C10:0	decanoic acid	1.92	0.44	6.76	30.34	5.43	80.72
C12:0	dodecanoic acid	0.25	0.00	0.65	4.20	0.00	10.75
C14:0	tetradecanoic acid	1.34	0.55	2.37	25.82	3.90	83.08
C14:1	myristoleic acid	0.13	0.00	2.63	2.04	0.00	37.91
C15:0	pentadecanoic acid	0.32	0.00	1.15	4.89	0.00	22.80
C16:0	palmitic acid	24.65	14.28	31.47	448.10	82.54	1141.83
C16:1	palmitoleic acid	1.39	0.57	3.27	24.47	3.16	111.98
C17:0	heptadecanoic acid	0.42	0.00	0.84	7.02	0.00	14.59
C18:0	stearic acid	16.92	7.37	32.30	334.42	45.22	756.68
C18:1n9	oleic acid (OA)	16.64	9.06	48.15	281.09	64.89	1475.92
C18-1trans11	trans-vaccenic acid (VA)	1.49	0.00	2.26	24.12	7.46	51.91
C18:2n6	linoleic acid (LA)	18.31	9.58	27.71	303.18	60.37	741.09
C18:3n6	gamma linolenic acid (GLA)	0.27	0.00	1.38	4.35	0.00	16.05
C18:3n3	α linolenic acid (ALA)	0.58	0.00	2.26	9.79	0.00	39.09
C18:4	Stearidonic acid	0.00	0.00	0.00	0.00	0.00	0.00
C20:4	arachidonic acid (ARA)	8.14	1.01	13.02	130.82	25.48	232.88
C20:5	eicosapentaenoic acid (EPA)	1.47	0.37	9.36	24.48	3.10	235.36
C22:0	behenic acid	0.31	0.00	2.10	3.38	0.00	39.96
C22:1cis13	erucic acid	0.00	0.00	0.00	0.00	0.00	0.00
C22:4n6	docosatetraenoic acid (DTA)	0.81	0.00	2.00	12.27	0.00	26.87
C22:5n3	docosapentaenoic acid (DPA)	1.30	0.40	3.43	20.74	2.78	53.15
C22:6n3	docosahexaenoic acid (DHA)	2.80	0.67	5.72	44.97	11.37	113.14
C23:0	tricosanoic acid	0.19	0.00	1.02	2.39	0.00	20.62
C24:1	nervonic acid	0.12	0.00	2.97	2.04	0.00	80.69
SFA	saturated fatty acids	46.32	24.02	67.65	860.56	153.60	2036.48
MUFA	monounsaturated fatty acids	19.77	10.95	50.73	333.77	78.82	1555.01
PUFA	poly-unsaturated fatty	33.68	20.98	46.80	550.59	141.61	1148.25
UFA	unsaturated fatty acids	53.45	32.35	75.75	884.36	220.43	2322.14
n3	Omega 3 fatty acids	6.15	3.08	11.39	99.98	28.99	286.47
n6	Omega 6 fatty acids	27.53	15.61	37.15	171.56	45.82	309.47
n9	Omega 9 fatty acids	1.61	0.70	3.88	26.16	7.46	105.06
